# TIde: a software for the systematic scanning of drug targets in kinetic network models

**DOI:** 10.1186/1471-2105-10-344

**Published:** 2009-10-19

**Authors:** Marvin Schulz, Barbara M Bakker, Edda Klipp

**Affiliations:** 1 Theoretical Biophysics, Institute for Biology, Humboldt Universität zu Berlin, Invadilenstraße. 42, 10115 Berlin, Germany; 2 Current address: Department of Pediatrics, Center for Liver, Digestive and Metabolic Diseases, University Medical Center Groningen, University of Groningen, Hanzeplein 1, NL-9713 GZ Groningen, the Netherlands; 3 Department of Molecular Cell Physiology, Faculty of Earth and Life Sciences, Vrije Universiteit Amsterdam, De Boelelaan 1085, 1081HV Amsterdam, the Netherlands

## Abstract

**Background:**

During the stages of the development of a potent drug candidate compounds can fail for several reasons. One of them, the efficacy of a candidate, can be estimated *in silico *if an appropriate ordinary differential equation model of the affected pathway is available. With such a model at hand it is also possible to detect reactions having a large effect on a certain variable such as a substance concentration.

**Results:**

We show an algorithm that systematically tests the influence of activators and inhibitors of different type and strength acting at different positions in the network. The effect on a quantity to be selected (e.g. a steady state flux or concentration) is calculated. Moreover, combinations of two inhibitors or one inhibitor and one activator targeting different network positions are analysed. Furthermore, we present TIde (Target Identification), an open source, platform independent tool to investigate ordinary differential equation models in the common systems biology markup language format. It automatically assigns the respectively altered kinetics to the inhibited or activated reactions, performs the necessary calculations, and provides a graphical output of the analysis results. For illustration, TIde is used to detect optimal inhibitor positions in simple branched networks, a signalling pathway, and a well studied model of glycolysis in *Trypanosoma brucei*.

**Conclusion:**

Using TIde, we show in the branched models under which conditions inhibitions in a certain pathway can affect a molecule concentrations in a different. In the signalling pathway we illuminate which inhibitions have an effect on the signalling characteristics of the last active kinase. Finally, we compare our set of best targets in the glycolysis model with a similar analysis showing the applicability of our tool.

## Background

In the current pharmaceutic development new drugs are often found by screening a library of small molecular entities (SME) against so-called 'blockbuster targets' which are supposed to play a relevant role in the onset of a certain disease. The development of drugs for new targets is in most cases less interesting for a pharmaceutical company due to the fact that the research is more expensive, they fail pre-clinical trials more often and are in most cases financially less successful [1,2]. In order to increase the productivity of 'Research and Development' (R&D) when focussing on novel targets a possible way is to identify candidates which are likely to fail trials earlier in the drug development process [3].

One problem that drugs against novel targets can cause is their possible lack of efficacy. During the development, possible targets are validated via knock-out experiments which work in a totally different way than medication with competitive inhibitors against the corresponding enzymes. While in the first case the flux through a certain pathway can be completely shut down, in the second case it will only be partially decreased which the system can overcome, e.g. by substrate accumulation or feedback regulation. Therefore quantitative modelling should be incorporated into drug research.

A systematic approach to the identification of possible drug targets in a reaction network renders possible with the established tools and methods used in systems biology. Over the last years more and more mathematical models for chemical reaction networks have proven to be successful in predicting an microorganism's response to changes in its environment and to perturbations in its gene expression [4-6]. These models are being collected in steadily growing databases like BioModels [7] or JWS online [8]. A promising approach to a systematic drug design is to simulate possible inhibitors to any reaction in a given network and to quantify their effects on a given observable. This observable can be defined as any inner variable of the system, e.g. the concentration of a substance or the flux through a certain reaction which is altered in the pathological state. For a more complex analysis this observable can also be defined as any function of these variables. From the time course of the observable, several characteristics can be extracted for later comparison. These include the steady state values, which are interesting for models of metabolic pathways, and several characteristics, which are relevant to signalling cascade models, namely the integrated concentration, the characteristic time, the signal duration, and the signal amplitude as described in [9,10]. Given this information from several simulations using different inhibition targets, types, and inhibitor concentrations, one can select favourable modification scenarios. "Favourable" means here that in a certain scenario a given observable reaches a desired value while the system is perturbed by few inhibitors in small concentrations.

Such a kind of analysis has already been applied manually to different kinds of small example models [11-14] and larger, biologically relevant models [15,16].

Other approaches to determine modified enzyme activities in order to achieve a certain change in a systems behaviour already exist (e.g. [17] and [18]), but none of them exhaustively searches the space of possible drug combinations, tries to minimise the amount of used inhibitor, or explicitly models different types of modification kinetics. A broad overview on similar methods can be found in [19].

## Implementation

**Figure 1:**
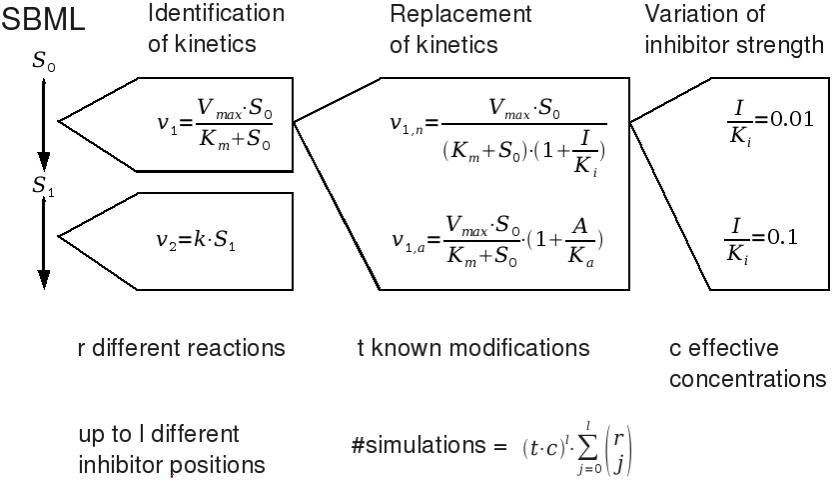
**Overview on the simulation steps performed in the algorithm**.

In order to simplify the error prone process of inhibiting or activating (from now on referred to as modifying) single reactions and investigating the results in a given ODE model, we have developed a platform independent tool written in Python [20] which performs this analysis automatically. The tool called TIde (Target Identification, ) works in three steps (as shown in Figure 1). First, it imports an ODE model given in the Systems Biology Markup Language (SBML) format [21] making use of libSBML [22]. The reaction kinetics in the imported model are identified by comparing their formulas to kinetic formulas from an internal database. This database is based on kinetics from the Systems Biology Ontology (SBO) [23] and was later extended manually. Second, it replaces individual reaction kinetics or combinations thereof by corresponding modifier kinetics and simulates the altered models for different modifier concentrations. Finally, it systematically compares the results of these simulations in order to determine single or multiple optimal drug targets.

In the following, these three steps of the tool's functionality will be explained.

### Identification of reaction kinetics

Starting from an SBML model, TIde is supposed to simulate the effects of different modifications of single reactions and combinations thereof. Therefore, the first step of the tool is the identification of the present kinetic formulas for each reaction by numerically comparing them to known kinetic types collected in an internal database. The identity of two formulas is judged by testing all possible parameter matchings of those formulas, repeatedly replacing the parameters by random values, and checking whether the results are similar except for numerical inaccuracy. Using the information about the present kinetics, the database can be searched for known modifier formulas. The identification of the kinetic parameters makes it possible to automatically replace a kinetic by a formula representing a certain kind of modification mechanism acting on the corresponding enzyme.

### Replacing reaction kinetics by corresponding modifications

Then, a set of new models is created in which systematically every reaction is replaced by a modification. During this step up to five new models per reaction can be created since four types of modifications are known to the database. These types are competitive, uncompetitive, and noncompetitive inhibition and nonessential activation. Additionally, a competitive inhibition for cofactors is available.

Unknown formulas (*v*_*i*_(*S*,*p*) with reaction velocity *v*_*i *_which is dependent on substance concentrations *S *and parameters *p*) will automatically be added to the internal database. Also a standard noncompetitive inhibition (, with inhibitor concentration *I *and its dissociation constant *K*_*i*_) and a standard nonessential activation () with activator concentration *A *and its dissociation constant *K*_*a*_) will be created. Because of that the number of new models created during this step will be at least twice as large as the number of reactions included in the original model.

In a step including user interaction, also different competitive and uncompetitive inhibitions can be included into the internal database. The user interaction involves the identification of *K*_*m *_values for substrates of reactions which are supposed to be inhibited. For the selected *K*_*m *_values new competitive inhibitions are created by multiplying the original *K*_*m *_by a factor . Uncompetitive inhibitions are created similarly by dividing the *K*_*m *_and V_*max *_values by the same factor. For SBML models including complex kinetics this step should be taken as the database can not cover all possible types of kinetic formulas.

TIde can also be used to check combinations of modifiers. These modifications do not need to be of the same type, but they have to affect different reactions.

### Comparing results of different modifications

In order to examine the effects of different modifications the user has to define the objective function which is to be maximized or minimized. This function can be an arbitrary formula containing substance concentrations or fluxes or a combination of them if the steady values are of interest (e.g. ). In case that signalling characteristics should be evaluated the objective function can in the current implementation only be a single compound concentration.

The effects of different inhibitor concentrations can be evaluated by either testing several distinct effective concentrations ( or ) in separate simulations or one simulation of a continuous titration of the inhibitor. Optionally these titrations can also be performed until the objective function has reached a certain value.

After the simulations of the new models have been performed making use of either the SciPy [24] library, the SBML Ode Solver library [25], or Copasi [26], the absolute differences between the original value of the objective function and its new values in the modification scenarios are calculated and displayed.

## Results and Discussion

We analysed four different models with the TIde tool (using Copasi for simulation) as discussed in the following. The first two models were simple metabolic pathways including a branch, the third was a simple signalling cascade, and the fourth one was a biologically relevant example of aerobic metabolism in *Trypanosoma brucei *[6].

### Prebranch model

**Figure 2:**
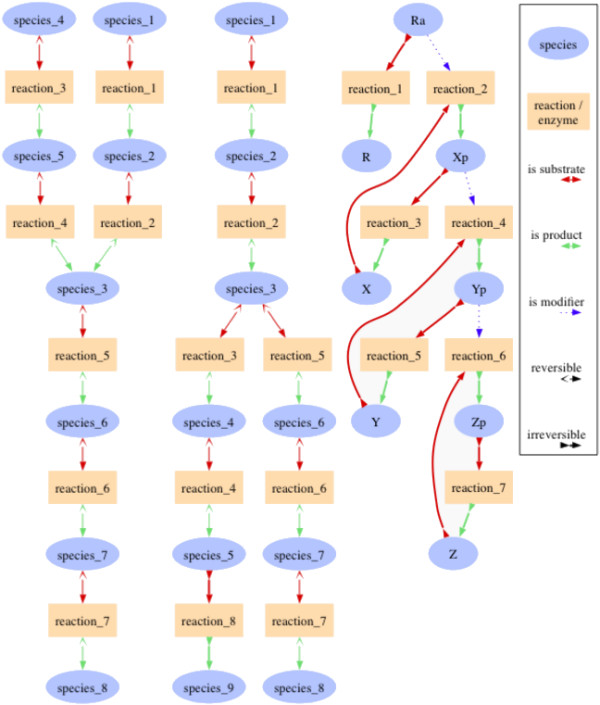
**Graphs from the prebranch, the postbranch, and the signalling cascade model**. The first two models contain only reversible Michaelis Menten kinetics with most parameters (*V*_*r *_(maximum backward velocity), *K*_*ms *_(binding constant of the substrate), *K*_*mr *_(binding constant of the product)) set to 1 and all *V*_*f *_(maximum forward velocity) values set to 10. The concentrations of the topmost substances in these models (species_1 and in the prebranch species_4) are set to 1 and kept fixed and the concentrations of the bottommost ones are kept at 0. In both cases the steady state concentration of species_7 is to be minimized. In the third model the concentrations of the activated receptor (*Ra*) and the inactive kinases (*X*, *Y*, and *Z*) are set to 1 while all others are set to 0. The phosphorylation steps (reactions 2, 4, and 6) occur at a rate of , the dephosphorylation steps (reactions 3, 5, and 7) at , and the receptor inactivation (reaction 1) at *v *= 0.1 * *Ra*. This figure has been produced with the help of sbml2dot [28].

The first two models (shown in Figure 2, left and middle) were extensions of a simple linear reaction chain by a small pathway bringing "biomass" either towards or away from the center of the chain. By this extension the pathway is divided into three subpathways. In the models we chose the concentration of species_7 as an objective function for minimization. With these models we did not only want to infer optimal modification targets in branched systems but also test whether it was possible (and favourable) to target enzymes in a certain pathway when a substance which is to be affected is in a different one. The result of the search for optimal drug targets depended on the parameters in the model. Nevertheless, in the scope of this article only the results for the most likely parameter set (see Figure 2) can be discussed in detail.

**Figure 3:**
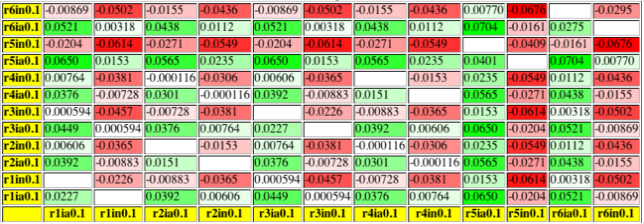
**Effects of different modifications on the prebranch model**. The axes of the tables specify the reaction which is modified, the modification type and the effective concentration of the modifier ( or ). Descriptions have been abbreviated in order to reduce the table size; *r3in0.1 *means that reaction_3 is noncompetitively inhibited with an effective inhibitor concentration of 0.1. Other possible types of modification are competitive inhibition (c), cofactor competitive inhibition (k), uncompetitve inhibition (u), or nonessential activation (a). Values on the diagonal describe the absolute change in the steady state concentration of species_7 when only one reaction is modified while values apart from the diagonal describe the change resulting from modifying two reactions at once. Near diagonal entries are blank because one single reaction is not modified twice.

As shown in the upper part of Figure 3, inhibiting a single reaction (5 or 6) in the same pathway as species_7 was more effective than inhibiting two reactions anywhere else. Because of the large equilibrium constants, which make "early" reactions in a linear pathway better targets, also combined inhibitions of reactions 1 and 5 or 3 and 5 have a quite strong effect. An inhibition of reactions 1 and 3 had in this case a larger effect than inhibiting only reaction 5. Still this combined inhibition in distant pathways was only slightly more advantageous than a single inhibition in the same pathway as the observed species. To the authors' surprise, at equilibrium constants equal to one (data not shown) it seemed to be quite ineffective to use modifications in other pathways in order to alter the concentration of a substance in a certain one, even if all reactions leading towards the observed substance were targeted. Nevertheless, a metabolic pathway in which the equilibrium constants are not bigger than one is very unlikely because it would not degrade the initial substance without the supply of external energy.

### Postbranch model

The postbranch model (see Figure 2, middle) was a slight modification of the previous model in which the third pathway was not directed to carry biomass into the center of the model but away from it. Here the idea was to study whether this third pathway could be activated in order to compete for biomass from the pathway including species_7.

**Figure 4:**
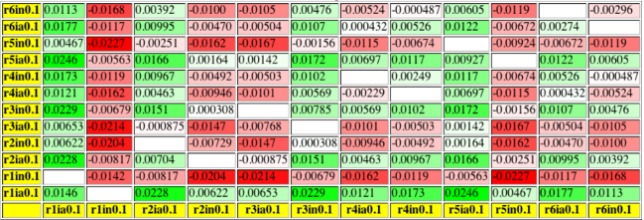
**Same analysis of the postbranch model as done in Figure 3**.

As shown in Figure 4, in this model it was most effective to inhibit reactions 1 and 5. Because of the large equilibrium constants the combination of inhibitions at reactions 1 and 2, which are optimal targets at constants equal to one (data not shown), was a little less effective while inhibiting reaction 1 and activating reaction 3 was a possible alternative. So, an activation of a competing pathway could not outperform other inhibitions but it made a good addition to them.

### Simple signalling cascade

The third model was a signalling cascade (see Figure 2, right). Here, we observed the effects of single modifications of the phosphorylation and dephosphorylation steps on the last activated kinase. Since the active receptor was constantly degraded, the steady state concentration of the last activated kinase was always zero. Therefore, we investigated more suitable characteristics of the dynamic behaviour (see Table 1). The integrated concentration, the characteristic time, the signal amplitude and the duration were influenced positively by the phosphorylation reactions, which means that an activation of the reaction increased the value of the signal characteristics. In the case of the dephosphorylation reactions the effect was adverse. In most cases the first reactions had a larger effect than the latter ones although some characteristics seemed to differ from that rule, e.g. the signal amplitude which could not be increased beyond a certain value in this model.

**Table 1:** Relative changes of the signalling characteristics of "Zp" in the small cascade model.

**Target**	**Amplitude**	**Integrated signal**	***σ***	***τ***
reaction_1	-0.02738	44.05890	14.57600	23.23040
reaction_2	-0.03430	-7.29018	-1.94723	-3.11361
reaction_3	0.04522	15.52320	4.26640	7.72552
reaction_4	-0.02467	-6.55773	-1.81591	-2.67058
reaction_5	0.02314	14.16874	4.16209	7.20750
reaction_6	-0.05883	-7.17961	-1.63696	-2.08881
reaction_7	-0.01467	10.28007	3.47335	5.24139

reference	1.57415	59.36095	18.85494	32.83275

The values in the table depict the relative changes of the characteristics after noncompetitive inhibition of a certain reaction by 50% compared to the reference state.

### Model of Trypanosoma glycolysis

Finally, in order to demonstrate the applicability of our tool, we investigated possible modification patterns in an updated model of the aerobic metabolism in *Trypanosoma brucei *[27] (BioModels BIOMD0000000211). For an older version of this model a similar analysis has already been performed manually [15]. The updated model contains new and more detailed information on enzyme kinetics under more realistic conditions and therefore these new results could lead to more promising drug targets against sleeping sickness. We have chosen the steady state flux through the upper glycolysis as the variable to be minimized by different modifications.

**Figure 5:**
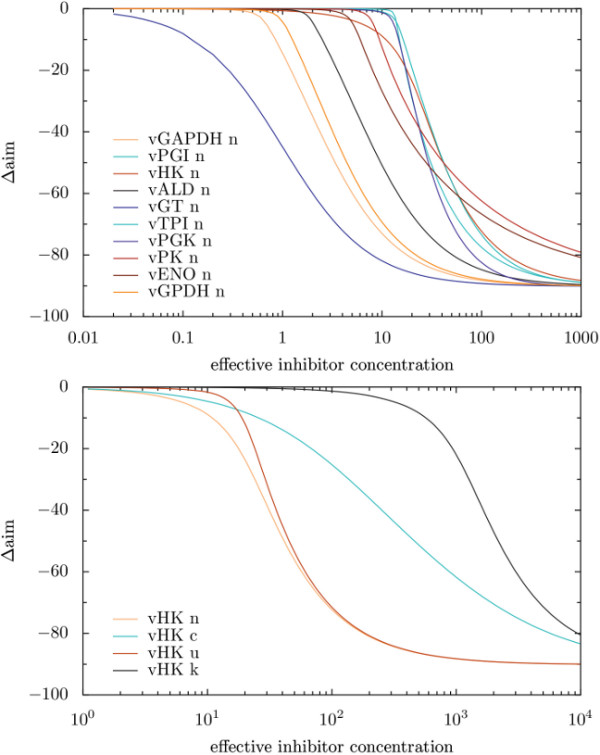
**Simulated titration of the modifier in the Trypanosoma model**. Depicted is the change in the objective function, which is the steady state flux through the upper glycolysis (reference flux ≈ 90). The first diagram depicts the effect of noncompetitive inhibitions in different positions in the network. In the second diagramm, effects of different inhibition types on the hexokinase are shown.

Since the data produced by TIde was too extensive to display in paperform, only specific results could be shown in Figure 5 and Tables 2 and 3. The favourability of inhibition target and kinetic type over others depended on the degree to which the flux through the glycolysis should be reduced in the parasite (see Figure 5). Therefore, we focussed on inhibitor concentrations decreasing the flux by 50% which was supposed to be sufficient to kill the parasite. As proposed earlier [15], the most promising target was the glucose transporter (GT). The best targets inside the glycolysis to achieve this goal were the phosphoglycerate mutase (PGM), the glyceraldehyde-3 phosphate dehydrogenase (GAPDH), the glycorol-3-phosphate dehydrogenase (GPDH), the enolase (ENO), and the fructose bisphosphate aldolase (ALD) in this order. As depicted in Table 1, also in this group the quality of a target depended on the type of inhibition. In the case that only competitive inhibitors would be available the GAPDH made an equally potent target as the GT. Earlier results favoured the targets ALD, GAPDH, phosphoglycerate kinase (PGK), and GPDH in this order besides the GT. These findings do not differ that much from the current results except for the PGK. This difference can be explained by different enzyme kinetics, the fact that ENO was not included as a single reaction in the old model, and a slightly different analysis. 

Another topic we wanted to investigate were synergisms and antagonisms of dual inhibitions in this model. The strongest of them are shown in Table 3. As already known, a strong synergism exists between the glycerol 3 phosphate oxidase (GPO) and the glycerol kinase (GK) but also other combinations yielded interesting results. A competitive inhibition of the triosephosphate isomerase gave a strong synergism together with a competitive inhibition for the cofactors for the phosphofructokinase (PFK), with inhibitions of GK, and with an activation of GPO. Strong antagonistic effects occurred between an activation of the hexokinase or the glucose transporter and competitive (also for cofactors) inhibitions of the phosphoglycerate kinase and PFK. The agreement of these results with the available literature and interesting new findings showed the  validity and utility of our tool. 

**Table 2:** Effective inhibitor concentrations necessary to kill Trypanosoma brucei.

	**noncompetitive**	**uncompetitive**	**competitive**
Glucose transport	1.09	1.27	6.13
Phosphoglycerate mutase	1.81	1.81	> 10000
Glyceraldehyde-3-p. dehydr.	2.89	5.32	6.17
Glycerol-3-p. dehydr.	3.79	3.88	253
Enolase	6.4	6.4	> 10000
Aldolase	8.14	8.36	319

Shown are the concentrations required to reduce the flux through the upper glycolysis by 50% for different inhibition types and targets.

**Table 3:** Strongest synergisms and antagonisms of combined modification in the Trypanosoma model.

**Modification 1**	**Modification 2**	**Effect 1**	**Effect 2**	**Combined effect**
GPO c/n/u	GK c/k/n/u	≈ -1.2	≈ -0.07	≈ -84
PFK k	TPI c	-0.026	-1.48	-90
PGK k	PGM c	-0.13	-0.37	-20
TPI c	GK c/k/n/u	-1.48	≈ 0.07	≈ 18.5
TPI c	GPO a	-1.48	-0.04	-16

GT a	PFK k	23	-0.026	-90
GT a	PGK k	23	-0.13	-12
HK a	PFK c	0.6	-9	-90

Numerical values are the relative flux reductions in  (reference flux: ≈ 90, corresponding to 1.5·10^-3 ^) resulting from a modification at an effective concentration of 100. The abbreviations stand for the targets (glycerol 3 phosphate oxidase (GPO), glycerol kinase (GK), phosphofructokinase (PFK), triosephosphate isomerase (TPI), phosphoglycerate kinase (PGK), and hexokinase (HK)) and the modification types (competitive (c), noncompetitive (n), uncompetitive (u), competitive for cofactors (k), and activation (a)).

## Conclusion

We have introduced an algorithm to systematically test the effect of modifications or modifier combinations depending their target and type as well as TIde, an implementation working on ODE models in the SBML format. This process can be understood as an extension of metabolic control analysis to multiple and strong perturbations in an ODE model. Although TIde seems a bit difficult to use in the first place needing a reliable ODE model, being a command line tool, and having a limited kinetic database the tool still has many advantages. Since TIde is not platform dependend it can be run on any kind of server making it possible to analyse even large models, due to the extendible kinetic database it can be applied to any ODE model, and because of the flexible objective function it can be used for many different purposes. Furthermore, we have tested our tool with a new version of the glycolysis in *Trypanosoma brucei*. Our results are except for some explainable changes in good agreement with older findings proving the applicability of TIde.

Given a reliable ODE model and the information our tool provides, the process of determining possible drugs is more directed into efficacy of the candidates in pre-clinical studies. Inclusion of this analysis will increase the probability of a candidate to become a potent drug, and decrease the cost of the development of 'new target' drugs. This fact will hopefully draw the attention of the pharmaceutical industry towards the results produced by systems biology. Since this research area is still growing more and more ODE models, which are a necessary input to our method, will become available.

In the future we plan to extend our tool to handle constraints which have to be fulfilled during simulation. The idea behind this is that certain modifications and effective modifier concentrations could lead to the death of the simulated organism (*e.g*. if the ATP concentration drops below a certain level). Such lethal modifications should be considered in order to identify possible side effects of the potential drug.

## Availability and requirements

• **Project name: **TIde

• **Project home page: **

• **Operating systems: **Cross platform

• **Programming language: **Python, C

• **Other requirements: **Python 2.5 or higher, SciPy 0.5 or higher, NumPy 1.1 or higher, PyX 0.9 or higher, semanticSBML 1.0 or higher, libSBML 3.3

• **License: **GNU GPL

• **Any restrictions to use by non-academics: **None

Furthermore, we have attached a walkthrough for our tool (see Additional file 1), its source code (see Additional file 2), and a Windows installer for the required tools and libraries (see Additional file 3) to the article.

## Authors' contributions

MS wrote the tool, conducted the computational experiments, and drafted the manuscript. BMB participated in the design of the tool. EK designed the tool and the numerical experiments and helped to draft the manuscript. All authors read and approved the final manuscript.

## Supplementary Material

Additional file 1 **Walkthrough**. Shows how the tool is used from the command line. Click here for file

Additional file 2 **TIde-1.2.1 source code**. Contains the packed python source code of our tool. Click here for file

Additional file 3 **TIde-1.2.1 prerequisites installer**. A Windows installer for all programs and libraries required by TIde. Click here for file
