# Supplementary material for: TIde: a software for the systematic scanning of drug targets in kinetic network models
Source: BMC Bioinformatics. 2009 Oct 19;10:344. doi: 10.1186/1471-2105-10-344 (PMC2773792; doi:10.1186/1471-2105-10-344)
Supplement: Additional file 1 — Walkthrough. Shows how the tool is used from the command line. [file 1471-2105-10-344-S1.PDF]

Walkthrough

Generating inhibition kinetics

In this section the handling of the single tools on a machine running Linux will be explained in detail. For this purpose the file BIOMD0000000211.xml (`http://www.ebi.ac.uk/biomodels/models-main/publ/BIOMD0000000211.xml`) has been downloaded from the BioModels database into the models subfolder. Since this model contains many not so common kinetics one has to include new inhibition kinetics to the internal database. For this purpose one calls the tool

```
python add_new_kinetics.py models/BIOMD0000000211.xml
```

With this tool the user selects the K_m values which are affected by the inhibitor in the corresponding inhibition kinetics. Two different sets of K_m values can be chosen. First, the values for the general substrates and products are selected. From this set the competitive and the uncompetitive inhibition kinetics are generated. Second, the values for the cofactors are selected. From this set only a competitive inhibition will be created. E.g. for the reaction vHK with the kinetics

$$\frac{compartment\_2 * Vmax\_v2 * \frac{species\_10}{KGlcInt\_v2} * \frac{species\_11}{KATPg\_v2}}{(1 + \frac{species\_11}{KATPg\_v2} + \frac{species\_12}{KADPg\_v2}) * (1 + \frac{species\_10}{KGlcInt\_v2} + \frac{species\_14}{KGlc6P\_v2})}$$

selecting the sets [KGlcInt_v2,KGlc6P_v2] and [KATPg_v2,KADPg_v2] will yield the new inhibition kinetics:

$$\frac{compartment\_2 * Vmax\_v2 * \frac{species\_10}{KGlcInt\_v2*(1+\frac{i}{ki})} * \frac{species\_11}{KATPg\_v2}}{(1 + \frac{species\_11}{KATPg\_v2} + \frac{species\_12}{KADPg\_v2}) * (1 + \frac{species\_10}{KGlcInt\_v2*(1+\frac{i}{ki})} + \frac{species\_14}{KGlc6P\_v2*(1+\frac{i}{ki})})}$$

(competitive),

$$\frac{compartment\_2 * Vmax\_v2 * \frac{species\_10*(1+\frac{i}{ki})}{KGlcInt\_v2} * \frac{species\_11}{KATPg\_v2}}{((1 + \frac{species\_11}{KATPg\_v2} + \frac{species\_12}{KADPg\_v2}) * (1 + \frac{species\_10*(1+\frac{i}{ki})}{KGlcInt\_v2} + \frac{species\_14*(1+\frac{i}{ki})}{KGlc6P\_v2})) * (1 + \frac{i}{ki})}$$

(uncompetitive), and

$$\frac{compartment\_2 * Vmax\_v2 * \frac{species\_10}{KGlcInt\_v2} * \frac{species\_11}{KATPg\_v2*(1+\frac{i}{ki})}}{(1 + \frac{species\_11}{KATPg\_v2*(1+\frac{i}{ki})} + \frac{species\_12}{KADPg\_v2*(1+\frac{i}{ki})}) * (1 + \frac{species\_10}{KGlcInt\_v2} + \frac{species\_14}{KGlc6P\_v2})}$$

(cofactor competitive).

After having added all inhibition kinetics to the internal database the actual computation can be started. Here three different types of computations can be started which will be explained in detail. Please be warned that due to the size of the model the analyses can take up to a week on a normal PC.

Discrete 2-dimensional analysis

Calling

```
python model.py -f models/BIOMD0000000211.xml -a vHK -i 1 -l 2
```

will start a discrete, 2-dimensional analysis on the model using up to 2 different inhibitors at an effective concentration of 1 and calculating the resulting difference in the flux through the upper glycolysis (reaction vHK). Another optional arguments for this tool is

```
-s
```

for signalling models which will force the tool to additionally evaluate signalling characteristics. In order to display the results as a table the user is able to call

```
python html.py models/BIOMD0000000211/sdh
```

which is creating the file models/BIOMD0000000211/sdh.html. An optional analysis that can be run is the search for synergistic or antagonistic effects of dual inhibitions/activations. The call

```
python sdhanalysis.py models/BIOMD0000000211/sdh -v 2 -t 70
```

will look for synergisms or antagonism which are seventy-fold stronger than expected from the sum of single effects and will output the results in a very verbose way.

Continuous analysis

Calling

```
python model.py -f models/BIOMD0000000211.xml -a vHK -i 100 -t
```

starts a continuous evaluation of the flux through the upper glycolysis for effective inhibitor concentrations up to 100. With the tool

```
python pdfplot.py models/BIOMD0000000211/sdh
```

two pdf files will be created (models/BIOMD0000000211/sdha.pdf and sdhi.pdf). With the optional arguments

```
-r
```

and

```
-x
```

the range of the x axis and a regular expression of the curve names which are to be displayed can be given.

Estimation of inhibitor concentrations

Calling

```
python model.py -f models/BIOMD0000000211.xml -a vHK -e 0.5
```

starts an estimation of how much effective inhibitor concentration is needed to fullfil a certain a certain goal, e.g. in this case the reduction of the flow through reaction vHK to 0.5 of its reference value. With the optional argument

```
-t
```

the estimation of the inhibitor concentration is replaced by a continuous inhibitor titration which stops when the goal is achieved. A sorted list of the effective inhibitor concentrations can then be calculated by calling

```
python htmllist.py models/BIOMD0000000211/sdh
```

. The resulting output file will again be models/BIOMD0000000211/sdh.html.
