# Supplementary material for: TIde: a software for the systematic scanning of drug targets in kinetic network models
Source: BMC Bioinformatics. 2009 Oct 19;10:344. doi: 10.1186/1471-2105-10-344 (PMC2773792; doi:10.1186/1471-2105-10-344)
Supplement: Additional file 2 — TIde-1.2.1 source code. Contains the packed python source code of our tool. [file 1471-2105-10-344-S2.ZIP › TIde-1.2.1/documentation/install.html~]

TIde

|  |
| --- |
| TIdeTide is a tool for the automatic identification of optimal drug targets in kinetic models based on ordinary differential equations. Give a model in the popular SBML format it will identify promising drug targets for different effective modifier concentrations. |
| DownloadsTIde 1.2.1   SBOS is a Linux live DVD containing a working installation of TIde. Just download the ISO image, burn it, and boot your computer from this DVD.  Windows installer for all required libraries. Note that TIde has to be downloaded separately. |
| RequirementsPython <3.0, ≥2.5, SciPy ≥0.5, NumPy (Numeric and NumArray might be needed depending on the SciPy version), semanticSBML, libSBML 3.x. |
| OptionalPyX (for pdf output)   Copasi 4.4, SOSlib 1.6 (as different ODE solvers)   ParallelPython 1.5.3 (for parallel computation, not documented). |
| InstallFirst, you have to install all the required tools and libraries from the Requirements section. Second, extract the tarball into a directory of your choice. Then download the Systems Biology Ontology into the TIde directory. You will now be able to do some configurations in the file `global_options`. If you are running Linux, then you will be able to set the solvertype to c and if you have additionally Copasi or SOSlib installed, then the you will also be able to set it to copasi or soslib. If you do not want to use any external simulation software, set the solver to scipy. In case you have ParallelPython installed, you can set the use\_parallel option to 1 and add available servers to the `servers` file. The lines in the file are statements of the form `ip,computer_name,number_of_cpus_to_be_used`. |
| How to useFor the analysis of a single file you have to call `python model.py -f path_to_model` in the TIde main directory including the options  | option | meaning | example | optional/mandatory | default | | --- | --- | --- | --- | --- | | -a | Observable, e.g. species\_1 which is accumulating in the pathological state | species\_1 | mandatory | | -s | Model is a signalling pathway |  | optional | | -i | Different effective inhibitor concentrations | 0.1,1,10 | optional | 0.1 | | -g | Use only certain reactions as possible modification targets | reaction\_0,reaction\_1 | optional | all | | -l | Use up to this many modifications simultaneously | 2 | optional | 1 | | -t | Do the computation by a simulated titration |  | optional |  | | -e | Estimate effective inhibitor concentrations to achieve a reduction of the aim value to this factor. | 0.1 | optional |  |    After all computations have been performed you can call different output methods, e.g. `html.py`, `htmllist.py`, or `pdfplot.py`. The first argument to the tools is the name of the output file which has to be placed in the folder named after the model which was created in the previous step. E.g. `python html.py models/simple_signalling_model/sdh` will create the file models/simple\_signalling\_model/sdh.html. The options for html.py are  | option | meaning | example | optional/mandatory | default | | --- | --- | --- | --- | --- | | -i | Ignore lines containing the strings or lacking the strings, e.g. show only combinations including reaction\_3 but ignore entries with the effective inhibitor concentration of 0.375. | ^reaction\_3,0.375 | optional | | -s | Ignore control/response coefficients in the output. |  | optional |    while the options for pdfplot.py are  | option | meaning | example | optional/mandatory | default | | --- | --- | --- | --- | --- | | -r | Range of the x axis | 1,100 | optional | | -x | Take only curves whichs name matches this regular expression | ".\*(1|5).\*n$" | optional | .\* |    Another interesting script for analysing data of a 2-dimensional scan for good inhibition targets is `sdhanalysis.py`. With its help synergisms and antagonisms in the inhibition results can be revealed. The command line options for this script are  | option | meaning | example | optional/mandatory | default | | --- | --- | --- | --- | --- | | -t | Threshold how much stronger a synergism has to be compared to the sum of single effects | 5 | optional | 1 | | -v | Verbosity | 1 | optional | 0 | |
| Questions?You can contact the author of this software under `marvin.schulz@biologie.hu-berlin.de`. |
