# Supplementary material for: TIde: a software for the systematic scanning of drug targets in kinetic network models
Source: BMC Bioinformatics. 2009 Oct 19;10:344. doi: 10.1186/1471-2105-10-344 (PMC2773792; doi:10.1186/1471-2105-10-344)
Supplement: Additional file 2 — TIde-1.2.1 source code. Contains the packed python source code of our tool. [file 1471-2105-10-344-S2.ZIP › TIde-1.2.1/documentation/htmlpy.html]

TIde output


TIde Output

| r6in0.1 | 0.000260 | -0.00460 | 0.000260 | -0.00460 | -0.00292 | -0.00127 | -0.00292 | -0.00127 | -0.000200 | -0.00414 |  | -0.00212 |
| r6ia0.1 | 0.00457 | -0.000623 | 0.00457 | -0.000623 | 0.00119 | 0.00290 | 0.00119 | 0.00290 | 0.00415 | -0.000200 | 0.00202 |  |
| r5in0.1 | 0.000260 | -0.00460 | 0.000260 | -0.00460 | -0.00292 | -0.00127 | -0.00292 | -0.00127 |  | -0.00212 |  |  |
| r5ia0.1 | 0.00457 | -0.000623 | 0.00457 | -0.000623 | 0.00119 | 0.00290 | 0.00119 | 0.00290 | 0.00202 |  |  |  |
| r4in0.1 | 0.00334 | -0.00171 | 0.00334 | -0.00171 | 8.09e-05 | 0.00168 |  | 0.000861 |  |  |  |  |
| r4ia0.1 | 0.00164 | -0.00337 | 0.00164 | -0.00337 | -0.00167 | 8.09e-05 | -0.000817 |  |  |  |  |  |
| r3in0.1 | 0.00334 | -0.00171 | 0.00334 | -0.00171 |  | 0.000861 |  |  |  |  |  |  |
| r3ia0.1 | 0.00164 | -0.00337 | 0.00164 | -0.00337 | -0.000817 |  |  |  |  |  |  |  |
| r2in0.1 | -0.000251 | -0.00497 |  | -0.00256 |  |  |  |  |  |  |  |  |
| r2ia0.1 | 0.00509 | -0.000251 | 0.00247 |  |  |  |  |  |  |  |  |  |
| r1in0.1 |  | -0.00256 |  |  |  |  |  |  |  |  |  |  |
| r1ia0.1 | 0.00247 |  |  |  |  |  |  |  |  |  |  |  |
|  | r1ia0.1 | r1in0.1 | r2ia0.1 | r2in0.1 | r3ia0.1 | r3in0.1 | r4ia0.1 | r4in0.1 | r5ia0.1 | r5in0.1 | r6ia0.1 | r6in0.1 |

  
Please note, that the links in the table are not connected to any files. They give only a detailed description on the current field.
