# Supplementary material for: TIde: a software for the systematic scanning of drug targets in kinetic network models
Source: BMC Bioinformatics. 2009 Oct 19;10:344. doi: 10.1186/1471-2105-10-344 (PMC2773792; doi:10.1186/1471-2105-10-344)
Supplement: Additional file 2 — TIde-1.2.1 source code. Contains the packed python source code of our tool. [file 1471-2105-10-344-S2.ZIP › TIde-1.2.1/documentation/htmllistpy.html]

TIde output

0  
0.1  
0.3  
1.0  
10.0  
3.0  
activation  
amplitude  
auc  
noncompetitive  
reaction\_1\_\_i  
reaction\_3\_\_i  
reaction\_4\_\_i  
reaction\_5\_\_i  
reaction\_6\_\_i  
reaction\_7\_\_i  
reaction\_8\_\_i  
sigma  
tau  

|  |  |  |  |
| --- | --- | --- | --- |
| -44.5995965144 | auc\_reaction\_7\_\_i | 10.0 | noncompetitive |
| -44.4174738306 | auc\_reaction\_8\_\_i | 10.0 | activation |
| -35.9614091073 | auc\_reaction\_1\_\_i | 10.0 | activation |
| -29.387291043 | auc\_reaction\_4\_\_i | 10.0 | activation |
| -29.0891273252 | auc\_reaction\_6\_\_i | 10.0 | activation |
| -28.813609209 | auc\_reaction\_1\_\_i | 3.0 | activation |
| -28.336174104 | auc\_reaction\_5\_\_i | 10.0 | noncompetitive |
| -28.0146834629 | auc\_reaction\_3\_\_i | 10.0 | noncompetitive |
| -20.1501037005 | auc\_reaction\_1\_\_i | 1.0 | activation |
| -19.132795938 | auc\_reaction\_8\_\_i | 3.0 | activation |
| -18.2416696274 | auc\_reaction\_7\_\_i | 3.0 | noncompetitive |
| -17.9325188727 | tau\_reaction\_1\_\_i | 10.0 | activation |
| -17.8638479986 | auc\_reaction\_4\_\_i | 3.0 | activation |
| -16.9307180543 | auc\_reaction\_6\_\_i | 3.0 | activation |
| -15.0326609096 | auc\_reaction\_3\_\_i | 3.0 | noncompetitive |
| -14.7318319201 | tau\_reaction\_1\_\_i | 3.0 | activation |
| -13.6675377898 | auc\_reaction\_5\_\_i | 3.0 | noncompetitive |
| -13.1074620692 | tau\_reaction\_4\_\_i | 10.0 | activation |
| -11.7166352276 | tau\_reaction\_6\_\_i | 10.0 | activation |
| -10.6183259339 | sigma\_reaction\_1\_\_i | 10.0 | activation |
| -10.4410531535 | tau\_reaction\_1\_\_i | 1.0 | activation |
| -10.1478594469 | tau\_reaction\_3\_\_i | 10.0 | noncompetitive |
| -9.80451948719 | auc\_reaction\_4\_\_i | 1.0 | activation |
| -9.60531198684 | auc\_reaction\_1\_\_i | 0.3 | activation |
| -9.46194648631 | auc\_reaction\_6\_\_i | 1.0 | activation |
| -8.79530534318 | sigma\_reaction\_1\_\_i | 3.0 | activation |
| -8.64922610225 | auc\_reaction\_8\_\_i | 1.0 | activation |
| -8.50328692889 | tau\_reaction\_4\_\_i | 3.0 | activation |
| -8.42888442674 | tau\_reaction\_8\_\_i | 10.0 | activation |
| -8.15043952327 | tau\_reaction\_5\_\_i | 10.0 | noncompetitive |
| -8.03395132252 | tau\_reaction\_6\_\_i | 3.0 | activation |
| -7.29017048941 | auc\_reaction\_3\_\_i | 1.0 | noncompetitive |
| -7.17954505713 | auc\_reaction\_7\_\_i | 1.0 | noncompetitive |
| -7.15604997857 | sigma\_reaction\_4\_\_i | 10.0 | activation |
| -6.8352146511 | sigma\_reaction\_6\_\_i | 10.0 | activation |
| -6.75396000356 | sigma\_reaction\_3\_\_i | 10.0 | noncompetitive |
| -6.74490453868 | tau\_reaction\_8\_\_i | 3.0 | activation |
| -6.5576238585 | auc\_reaction\_5\_\_i | 1.0 | noncompetitive |
| -6.43574829271 | sigma\_reaction\_5\_\_i | 10.0 | noncompetitive |
| -6.35052930476 | sigma\_reaction\_1\_\_i | 1.0 | activation |
| -6.13044922163 | tau\_reaction\_3\_\_i | 3.0 | noncompetitive |
| -5.12856510426 | tau\_reaction\_5\_\_i | 3.0 | noncompetitive |
| -5.01674302152 | tau\_reaction\_1\_\_i | 0.3 | activation |
| -4.73770242319 | tau\_reaction\_4\_\_i | 1.0 | activation |
| -4.62100710783 | tau\_reaction\_6\_\_i | 1.0 | activation |
| -4.5453497112 | sigma\_reaction\_4\_\_i | 3.0 | activation |
| -4.46512716343 | tau\_reaction\_7\_\_i | 10.0 | noncompetitive |
| -4.4549543793 | sigma\_reaction\_6\_\_i | 3.0 | activation |
| -4.41444895846 | sigma\_reaction\_8\_\_i | 10.0 | activation |
| -4.07776388929 | auc\_reaction\_4\_\_i | 0.3 | activation |
| -4.0192607887 | sigma\_reaction\_7\_\_i | 10.0 | noncompetitive |
| -3.98273609337 | sigma\_reaction\_8\_\_i | 3.0 | activation |
| -3.9580726353 | auc\_reaction\_6\_\_i | 0.3 | activation |
| -3.9188358335 | sigma\_reaction\_3\_\_i | 3.0 | noncompetitive |
| -3.82931309643 | auc\_reaction\_1\_\_i | 0.1 | activation |
| -3.73408426143 | tau\_reaction\_8\_\_i | 1.0 | activation |
| -3.67064768204 | sigma\_reaction\_5\_\_i | 3.0 | noncompetitive |
| -3.57746323359 | tau\_reaction\_7\_\_i | 3.0 | noncompetitive |
| -3.40506305488 | reaction\_1\_\_i | 0 | activation |
| -3.4050290046 | reaction\_3\_\_i | 0 | noncompetitive |
| -3.34118338071 | sigma\_reaction\_7\_\_i | 3.0 | noncompetitive |
| -3.32359290026 | auc\_reaction\_8\_\_i | 0.3 | activation |
| -3.11360515001 | tau\_reaction\_3\_\_i | 1.0 | noncompetitive |
| -3.09584230022 | sigma\_reaction\_1\_\_i | 0.3 | activation |
| -2.73344400869 | auc\_reaction\_3\_\_i | 0.3 | noncompetitive |
| -2.67052721572 | tau\_reaction\_5\_\_i | 1.0 | noncompetitive |
| -2.54844640003 | sigma\_reaction\_6\_\_i | 1.0 | activation |
| -2.52721050981 | sigma\_reaction\_4\_\_i | 1.0 | activation |
| -2.52145006327 | auc\_reaction\_7\_\_i | 0.3 | noncompetitive |
| -2.46194552663 | auc\_reaction\_5\_\_i | 0.3 | noncompetitive |
| -2.28321245441 | sigma\_reaction\_8\_\_i | 1.0 | activation |
| -2.08877937136 | tau\_reaction\_7\_\_i | 1.0 | noncompetitive |
| -2.00515903272 | tau\_reaction\_1\_\_i | 0.1 | activation |
| -1.98321207975 | tau\_reaction\_4\_\_i | 0.3 | activation |
| -1.9559625512 | tau\_reaction\_6\_\_i | 0.3 | activation |
| -1.94722347973 | sigma\_reaction\_3\_\_i | 1.0 | noncompetitive |
| -1.81588452949 | sigma\_reaction\_5\_\_i | 1.0 | noncompetitive |
| -1.63693826995 | sigma\_reaction\_7\_\_i | 1.0 | noncompetitive |
| -1.55296632658 | auc\_reaction\_4\_\_i | 0.1 | activation |
| -1.5322620526 | tau\_reaction\_8\_\_i | 0.3 | activation |
| -1.50376457412 | auc\_reaction\_6\_\_i | 0.1 | activation |
| -1.24335130534 | sigma\_reaction\_1\_\_i | 0.1 | activation |
| -1.22737470599 | auc\_reaction\_8\_\_i | 0.1 | activation |
| -1.19509776578 | tau\_reaction\_3\_\_i | 0.3 | noncompetitive |
| -1.08324206153 | sigma\_reaction\_6\_\_i | 0.3 | activation |
| -1.07665624663 | amplitude\_reaction\_7\_\_i | 10.0 | noncompetitive |
| -1.05891716854 | sigma\_reaction\_4\_\_i | 0.3 | activation |
| -1.05673551696 | amplitude\_reaction\_8\_\_i | 10.0 | activation |
| -1.03788650144 | tau\_reaction\_5\_\_i | 0.3 | noncompetitive |
| -0.989523149521 | auc\_reaction\_3\_\_i | 0.1 | noncompetitive |
| -0.954601100165 | sigma\_reaction\_8\_\_i | 0.3 | activation |
| -0.898156600143 | auc\_reaction\_7\_\_i | 0.1 | noncompetitive |
| -0.893373251883 | auc\_reaction\_5\_\_i | 0.1 | noncompetitive |
| -0.832227697825 | tau\_reaction\_7\_\_i | 0.3 | noncompetitive |
| -0.757300955511 | tau\_reaction\_4\_\_i | 0.1 | activation |
| -0.746456685656 | tau\_reaction\_6\_\_i | 0.1 | activation |
| -0.735876810742 | sigma\_reaction\_3\_\_i | 0.3 | noncompetitive |
| -0.687261482577 | sigma\_reaction\_5\_\_i | 0.3 | noncompetitive |
| -0.614300872691 | sigma\_reaction\_7\_\_i | 0.3 | noncompetitive |
| -0.577511298883 | tau\_reaction\_8\_\_i | 0.1 | activation |
| -0.436350874347 | tau\_reaction\_3\_\_i | 0.1 | noncompetitive |
| -0.415001643124 | sigma\_reaction\_6\_\_i | 0.1 | activation |
| -0.404999945749 | sigma\_reaction\_4\_\_i | 0.1 | activation |
| -0.380911284507 | tau\_reaction\_5\_\_i | 0.1 | noncompetitive |
| -0.375700592256 | amplitude\_reaction\_8\_\_i | 10.0 | noncompetitive |
| -0.362742769573 | sigma\_reaction\_8\_\_i | 0.1 | activation |
| -0.325083692472 | amplitude\_reaction\_5\_\_i | 10.0 | noncompetitive |
| -0.314893517148 | amplitude\_reaction\_6\_\_i | 10.0 | activation |
| -0.307380910273 | tau\_reaction\_7\_\_i | 0.1 | noncompetitive |
| -0.293102353323 | amplitude\_reaction\_4\_\_i | 10.0 | activation |
| -0.278954031208 | amplitude\_reaction\_3\_\_i | 10.0 | noncompetitive |
| -0.267105884436 | sigma\_reaction\_3\_\_i | 0.1 | noncompetitive |
| -0.24987791682 | sigma\_reaction\_5\_\_i | 0.1 | noncompetitive |
| -0.248896899381 | amplitude\_reaction\_7\_\_i | 3.0 | noncompetitive |
| -0.223058132648 | sigma\_reaction\_7\_\_i | 0.1 | noncompetitive |
| -0.221687981058 | amplitude\_reaction\_8\_\_i | 3.0 | activation |
| -0.153690549035 | amplitude\_reaction\_1\_\_i | 10.0 | activation |
| -0.124173633462 | amplitude\_reaction\_4\_\_i | 3.0 | activation |
| -0.111574134849 | amplitude\_reaction\_6\_\_i | 10.0 | noncompetitive |
| -0.10087535205 | amplitude\_reaction\_6\_\_i | 3.0 | activation |
| -0.100553357763 | amplitude\_reaction\_8\_\_i | 3.0 | noncompetitive |
| -0.0902179557867 | amplitude\_reaction\_3\_\_i | 3.0 | noncompetitive |
| -0.0695210147738 | amplitude\_reaction\_5\_\_i | 3.0 | noncompetitive |
| -0.062241820933 | amplitude\_reaction\_1\_\_i | 10.0 | noncompetitive |
| -0.0588332522601 | amplitude\_reaction\_7\_\_i | 1.0 | noncompetitive |
| -0.056594369225 | amplitude\_reaction\_4\_\_i | 1.0 | activation |
| -0.0558361198049 | amplitude\_reaction\_1\_\_i | 3.0 | activation |
| -0.048050089585 | amplitude\_reaction\_1\_\_i | 3.0 | noncompetitive |
| -0.0441138448698 | amplitude\_reaction\_6\_\_i | 1.0 | activation |
| -0.0440810726197 | amplitude\_reaction\_8\_\_i | 1.0 | activation |
| -0.0342960474519 | amplitude\_reaction\_3\_\_i | 1.0 | noncompetitive |
| -0.0273799275081 | amplitude\_reaction\_1\_\_i | 1.0 | noncompetitive |
| -0.0246692759007 | amplitude\_reaction\_5\_\_i | 1.0 | noncompetitive |
| -0.0209029843643 | amplitude\_reaction\_4\_\_i | 0.3 | activation |
| -0.0161027755521 | amplitude\_reaction\_7\_\_i | 0.3 | noncompetitive |
| -0.0154095100363 | amplitude\_reaction\_6\_\_i | 0.3 | activation |
| -0.0146686026987 | amplitude\_reaction\_8\_\_i | 1.0 | noncompetitive |
| -0.0117337803863 | amplitude\_reaction\_4\_\_i | 10.0 | noncompetitive |
| -0.0114985605936 | amplitude\_reaction\_3\_\_i | 0.3 | noncompetitive |
| -0.00956695193798 | amplitude\_reaction\_1\_\_i | 0.3 | noncompetitive |
| -0.00888882626323 | amplitude\_reaction\_8\_\_i | 0.3 | activation |
| -0.00820806682526 | amplitude\_reaction\_5\_\_i | 0.3 | noncompetitive |
| -0.0075313761387 | amplitude\_reaction\_4\_\_i | 0.1 | activation |
| -0.00626791496768 | amplitude\_reaction\_1\_\_i | 1.0 | activation |
| -0.00534754077803 | amplitude\_reaction\_6\_\_i | 0.1 | activation |
| -0.00525721992877 | amplitude\_reaction\_7\_\_i | 0.1 | noncompetitive |
| -0.00399709986322 | amplitude\_reaction\_3\_\_i | 0.1 | noncompetitive |
| -0.00309092830588 | amplitude\_reaction\_1\_\_i | 0.1 | noncompetitive |
| -0.00286705920658 | amplitude\_reaction\_5\_\_i | 0.1 | noncompetitive |
| -0.00230781084791 | amplitude\_reaction\_8\_\_i | 0.1 | activation |
| -0.00109742048994 | amplitude\_reaction\_6\_\_i | 3.0 | noncompetitive |
| -5.73377926929e-14 | reaction\_1\_\_i | 1.0 | noncompetitive |
| -4.17521639081e-14 | reaction\_7\_\_i | 0.1 | activation |
| -2.95998871524e-14 | reaction\_3\_\_i | 10.0 | noncompetitive |
| -1.42327443057e-14 | reaction\_6\_\_i | 1.0 | noncompetitive |
| -1.22953776018e-14 | reaction\_3\_\_i | 0.3 | activation |
| -6.58295412028e-15 | reaction\_8\_\_i | 3.0 | noncompetitive |
| -5.41346367841e-15 | reaction\_4\_\_i | 0.1 | noncompetitive |
| -4.4980712165e-15 | reaction\_3\_\_i | 3.0 | activation |
| -2.23033423534e-15 | reaction\_8\_\_i | 3.0 | activation |
| -1.46968532011e-15 | reaction\_4\_\_i | 1.0 | activation |
| -7.10127077983e-16 | reaction\_5\_\_i | 0.1 | noncompetitive |
| -3.5402814821e-16 | reaction\_4\_\_i | 0.1 | activation |
| -3.31313732598e-16 | reaction\_4\_\_i | 3.0 | noncompetitive |
| -3.19376307506e-16 | reaction\_7\_\_i | 3.0 | activation |
| -3.18941687642e-16 | reaction\_5\_\_i | 0.3 | activation |
| -3.18840968608e-16 | reaction\_4\_\_i | 0.3 | activation |
| -3.15106280114e-16 | reaction\_8\_\_i | 0.3 | activation |
| -3.1087188919e-16 | reaction\_7\_\_i | 10.0 | activation |
| -3.09709070534e-16 | reaction\_5\_\_i | 3.0 | noncompetitive |
| -2.96601965628e-16 | reaction\_8\_\_i | 0.1 | activation |
| -2.93758067368e-16 | reaction\_1\_\_i | 0.1 | noncompetitive |
| -2.88944999218e-16 | reaction\_7\_\_i | 0.1 | noncompetitive |
| -2.84323668686e-16 | reaction\_5\_\_i | 10.0 | noncompetitive |
| -2.70444088108e-16 | reaction\_7\_\_i | 3.0 | noncompetitive |
| -2.63564491152e-16 | reaction\_3\_\_i | 10.0 | activation |
| -2.59335797572e-16 | reaction\_8\_\_i | 0.1 | noncompetitive |
| -2.57023539147e-16 | reaction\_4\_\_i | 1.0 | noncompetitive |
| -2.56383683001e-16 | reaction\_8\_\_i | 1.0 | noncompetitive |
| -2.53037586971e-16 | reaction\_3\_\_i | 1.0 | noncompetitive |
| -2.46678180319e-16 | reaction\_7\_\_i | 1.0 | activation |
| -2.46663892651e-16 | reaction\_7\_\_i | 1.0 | noncompetitive |
| -2.42296036502e-16 | reaction\_3\_\_i | 0.1 | activation |
| -2.42028951827e-16 | reaction\_3\_\_i | 3.0 | noncompetitive |
| -2.39497363427e-16 | reaction\_7\_\_i | 0.3 | noncompetitive |
| -2.34971640595e-16 | reaction\_3\_\_i | 1.0 | activation |
| -2.31200586577e-16 | reaction\_6\_\_i | 0.3 | noncompetitive |
| -2.2960834303e-16 | reaction\_5\_\_i | 0.3 | noncompetitive |
| -2.27662290955e-16 | reaction\_5\_\_i | 1.0 | activation |
| -2.26804080335e-16 | reaction\_7\_\_i | 10.0 | noncompetitive |
| -2.26594161914e-16 | reaction\_1\_\_i | 0.3 | activation |
| -2.26347117123e-16 | reaction\_5\_\_i | 3.0 | activation |
| -2.22585221538e-16 | reaction\_8\_\_i | 10.0 | activation |
| -2.20356545705e-16 | reaction\_6\_\_i | 1.0 | activation |
| -2.18943217133e-16 | reaction\_4\_\_i | 0.3 | noncompetitive |
| -2.14037584178e-16 | reaction\_6\_\_i | 3.0 | noncompetitive |
| -2.13635898829e-16 | reaction\_3\_\_i | 0.1 | noncompetitive |
| -2.08399183679e-16 | reaction\_6\_\_i | 10.0 | activation |
| -1.90223264505e-16 | reaction\_1\_\_i | 0.1 | activation |
| -1.87293401188e-16 | reaction\_8\_\_i | 1.0 | activation |
| -1.83531077946e-16 | reaction\_7\_\_i | 0.3 | activation |
| -1.57776396458e-16 | reaction\_5\_\_i | 0.1 | activation |
| -1.52365113755e-16 | reaction\_4\_\_i | 10.0 | activation |
| 0.0 | reaction\_4\_\_i | 0 | noncompetitive |
| 0.0 | reaction\_4\_\_i | 0 | activation |
| 0.0 | reaction\_5\_\_i | 0 | noncompetitive |
| 0.0 | reaction\_5\_\_i | 0 | activation |
| 0.0 | reaction\_6\_\_i | 0 | noncompetitive |
| 0.0 | reaction\_6\_\_i | 0 | activation |
| 0.0 | reaction\_7\_\_i | 0 | noncompetitive |
| 0.0 | reaction\_7\_\_i | 0 | activation |
| 0.0 | reaction\_8\_\_i | 0 | noncompetitive |
| 0.0 | reaction\_8\_\_i | 0 | activation |
| 3.31383363115e-16 | reaction\_5\_\_i | 1.0 | noncompetitive |
| 7.36887637458e-16 | reaction\_1\_\_i | 0.3 | noncompetitive |
| 1.92402613516e-15 | reaction\_6\_\_i | 3.0 | activation |
| 4.52790057581e-15 | reaction\_6\_\_i | 0.1 | activation |
| 7.47516361293e-15 | reaction\_5\_\_i | 10.0 | activation |
| 1.1783050136e-14 | reaction\_3\_\_i | 0.3 | noncompetitive |
| 2.4125741125e-14 | reaction\_8\_\_i | 0.3 | noncompetitive |
| 5.31102793859e-14 | reaction\_1\_\_i | 10.0 | noncompetitive |
| 7.23193241957e-14 | reaction\_6\_\_i | 0.3 | activation |
| 7.72103988268e-14 | reaction\_4\_\_i | 3.0 | activation |
| 1.4286949697e-13 | reaction\_6\_\_i | 0.1 | noncompetitive |
| 2.98860445799e-11 | reaction\_8\_\_i | 10.0 | noncompetitive |
| 6.49957081614e-11 | reaction\_6\_\_i | 10.0 | noncompetitive |
| 1.40966759511e-10 | reaction\_4\_\_i | 10.0 | noncompetitive |
| 5.02453121562e-10 | reaction\_1\_\_i | 3.0 | noncompetitive |
| 1.20221131686e-09 | reaction\_1\_\_i | 10.0 | activation |
| 9.06860240121e-09 | reaction\_1\_\_i | 3.0 | activation |
| 1.61235137027e-06 | reaction\_1\_\_i | 1.0 | activation |
| 0.00114469960559 | amplitude\_reaction\_8\_\_i | 0.3 | noncompetitive |
| 0.00129828525723 | amplitude\_reaction\_8\_\_i | 0.1 | noncompetitive |
| 0.00241676127038 | amplitude\_reaction\_1\_\_i | 0.1 | activation |
| 0.00275718913267 | amplitude\_reaction\_5\_\_i | 0.1 | activation |
| 0.00380807129627 | amplitude\_reaction\_3\_\_i | 0.1 | activation |
| 0.0044837189315 | amplitude\_reaction\_1\_\_i | 0.3 | activation |
| 0.00470435028936 | amplitude\_reaction\_7\_\_i | 0.1 | activation |
| 0.00498654084746 | amplitude\_reaction\_6\_\_i | 0.1 | noncompetitive |
| 0.00736519887919 | amplitude\_reaction\_5\_\_i | 0.3 | activation |
| 0.00741897768738 | amplitude\_reaction\_4\_\_i | 0.1 | noncompetitive |
| 0.01006250905 | amplitude\_reaction\_3\_\_i | 0.3 | activation |
| 0.0118489487688 | amplitude\_reaction\_7\_\_i | 0.3 | activation |
| 0.0126374030328 | amplitude\_reaction\_6\_\_i | 0.3 | noncompetitive |
| 0.018246229399 | amplitude\_reaction\_5\_\_i | 1.0 | activation |
| 0.0199386244493 | amplitude\_reaction\_4\_\_i | 0.3 | noncompetitive |
| 0.0231405201899 | amplitude\_reaction\_6\_\_i | 1.0 | noncompetitive |
| 0.0240760369785 | amplitude\_reaction\_3\_\_i | 1.0 | activation |
| 0.0257365683034 | amplitude\_reaction\_7\_\_i | 1.0 | activation |
| 0.0336290848099 | amplitude\_reaction\_5\_\_i | 3.0 | activation |
| 0.0409514785505 | amplitude\_reaction\_7\_\_i | 3.0 | activation |
| 0.0417492981747 | amplitude\_reaction\_3\_\_i | 3.0 | activation |
| 0.0440749718527 | amplitude\_reaction\_4\_\_i | 3.0 | noncompetitive |
| 0.0452247431129 | amplitude\_reaction\_4\_\_i | 1.0 | noncompetitive |
| 0.0522245126635 | amplitude\_reaction\_5\_\_i | 10.0 | activation |
| 0.05832219631 | amplitude\_reaction\_7\_\_i | 10.0 | activation |
| 0.0603319040923 | amplitude\_reaction\_3\_\_i | 10.0 | activation |
| 0.22333611757 | sigma\_reaction\_7\_\_i | 0.1 | activation |
| 0.250234929235 | sigma\_reaction\_5\_\_i | 0.1 | activation |
| 0.266819919158 | sigma\_reaction\_3\_\_i | 0.1 | activation |
| 0.312860914623 | tau\_reaction\_7\_\_i | 0.1 | activation |
| 0.383864242651 | sigma\_reaction\_8\_\_i | 0.1 | noncompetitive |
| 0.385286598115 | tau\_reaction\_5\_\_i | 0.1 | activation |
| 0.43411584037 | sigma\_reaction\_4\_\_i | 0.1 | noncompetitive |
| 0.438759117585 | tau\_reaction\_3\_\_i | 0.1 | activation |
| 0.443681628378 | sigma\_reaction\_6\_\_i | 0.1 | noncompetitive |
| 0.604825564424 | tau\_reaction\_8\_\_i | 0.1 | noncompetitive |
| 0.616125673024 | sigma\_reaction\_7\_\_i | 0.3 | activation |
| 0.689987353479 | sigma\_reaction\_5\_\_i | 0.3 | activation |
| 0.733778503127 | sigma\_reaction\_3\_\_i | 0.3 | activation |
| 0.793127001174 | tau\_reaction\_6\_\_i | 0.1 | noncompetitive |
| 0.808920837604 | tau\_reaction\_4\_\_i | 0.1 | noncompetitive |
| 0.873725616319 | tau\_reaction\_7\_\_i | 0.3 | activation |
| 0.882629807875 | auc\_reaction\_7\_\_i | 0.1 | activation |
| 0.893166773678 | auc\_reaction\_5\_\_i | 0.1 | activation |
| 0.985662052241 | auc\_reaction\_3\_\_i | 0.1 | activation |
| 1.07103758172 | tau\_reaction\_5\_\_i | 0.3 | activation |
| 1.1156802645 | sigma\_reaction\_8\_\_i | 0.3 | noncompetitive |
| 1.21334434088 | tau\_reaction\_3\_\_i | 0.3 | activation |
| 1.25847337449 | auc\_reaction\_8\_\_i | 0.1 | noncompetitive |
| 1.28333480114 | sigma\_reaction\_4\_\_i | 0.3 | noncompetitive |
| 1.30226131033 | sigma\_reaction\_6\_\_i | 0.3 | noncompetitive |
| 1.39496396633 | sigma\_reaction\_1\_\_i | 0.1 | noncompetitive |
| 1.58930779281 | auc\_reaction\_6\_\_i | 0.1 | noncompetitive |
| 1.64414347541 | sigma\_reaction\_7\_\_i | 1.0 | activation |
| 1.65293508596 | auc\_reaction\_4\_\_i | 0.1 | noncompetitive |
| 1.73989308624 | tau\_reaction\_8\_\_i | 0.3 | noncompetitive |
| 1.83250548236 | sigma\_reaction\_5\_\_i | 1.0 | activation |
| 1.93412758234 | sigma\_reaction\_3\_\_i | 1.0 | activation |
| 2.23961029519 | tau\_reaction\_1\_\_i | 0.1 | noncompetitive |
| 2.31164035146 | tau\_reaction\_6\_\_i | 0.3 | noncompetitive |
| 2.37923240148 | tau\_reaction\_4\_\_i | 0.3 | noncompetitive |
| 2.39147277607 | tau\_reaction\_7\_\_i | 1.0 | activation |
| 2.40116888676 | auc\_reaction\_7\_\_i | 0.3 | activation |
| 2.4601889574 | auc\_reaction\_5\_\_i | 0.3 | activation |
| 2.70437504635 | auc\_reaction\_3\_\_i | 0.3 | activation |
| 2.89674152877 | tau\_reaction\_5\_\_i | 1.0 | activation |
| 3.23956943395 | tau\_reaction\_3\_\_i | 1.0 | activation |
| 3.36360321029 | sigma\_reaction\_7\_\_i | 3.0 | activation |
| 3.40502900463 | reaction\_1\_\_i | 0 | noncompetitive |
| 3.40506305488 | reaction\_3\_\_i | 0 | activation |
| 3.47338897734 | sigma\_reaction\_8\_\_i | 1.0 | noncompetitive |
| 3.55821322817 | auc\_reaction\_8\_\_i | 0.3 | noncompetitive |
| 3.70057417688 | sigma\_reaction\_5\_\_i | 3.0 | activation |
| 3.85903711675 | sigma\_reaction\_3\_\_i | 3.0 | activation |
| 4.16208250864 | sigma\_reaction\_6\_\_i | 1.0 | noncompetitive |
| 4.24818204512 | sigma\_reaction\_1\_\_i | 0.3 | noncompetitive |
| 4.26639017881 | sigma\_reaction\_4\_\_i | 1.0 | noncompetitive |
| 4.26657881014 | auc\_reaction\_1\_\_i | 0.1 | noncompetitive |
| 4.60937348436 | auc\_reaction\_6\_\_i | 0.3 | noncompetitive |
| 4.84337629805 | auc\_reaction\_4\_\_i | 0.3 | noncompetitive |
| 5.04725567225 | tau\_reaction\_7\_\_i | 3.0 | activation |
| 5.24146091485 | tau\_reaction\_8\_\_i | 1.0 | noncompetitive |
| 5.98286888749 | tau\_reaction\_5\_\_i | 3.0 | activation |
| 6.0167702341 | sigma\_reaction\_7\_\_i | 10.0 | activation |
| 6.23140177055 | auc\_reaction\_7\_\_i | 1.0 | activation |
| 6.48046799546 | sigma\_reaction\_5\_\_i | 10.0 | activation |
| 6.52420547539 | auc\_reaction\_5\_\_i | 1.0 | activation |
| 6.56461953423 | tau\_reaction\_3\_\_i | 3.0 | activation |
| 6.66923843756 | sigma\_reaction\_3\_\_i | 10.0 | activation |
| 6.80009344355 | tau\_reaction\_1\_\_i | 0.3 | noncompetitive |
| 7.09024252638 | auc\_reaction\_3\_\_i | 1.0 | activation |
| 7.20748547827 | tau\_reaction\_6\_\_i | 1.0 | noncompetitive |
| 7.72549887004 | tau\_reaction\_4\_\_i | 1.0 | noncompetitive |
| 9.35438587945 | tau\_reaction\_7\_\_i | 10.0 | activation |
| 9.81927373841 | sigma\_reaction\_8\_\_i | 3.0 | noncompetitive |
| 10.2802097164 | auc\_reaction\_8\_\_i | 1.0 | noncompetitive |
| 10.7139803318 | tau\_reaction\_5\_\_i | 10.0 | activation |
| 11.4988510617 | tau\_reaction\_3\_\_i | 10.0 | activation |
| 12.1534213254 | sigma\_reaction\_6\_\_i | 3.0 | noncompetitive |
| 12.4093824305 | auc\_reaction\_7\_\_i | 3.0 | activation |
| 12.9324857085 | auc\_reaction\_1\_\_i | 0.3 | noncompetitive |
| 13.167545812 | auc\_reaction\_5\_\_i | 3.0 | activation |
| 13.6123414419 | sigma\_reaction\_4\_\_i | 3.0 | noncompetitive |
| 13.7364769582 | tau\_reaction\_8\_\_i | 3.0 | noncompetitive |
| 14.0459761613 | auc\_reaction\_3\_\_i | 3.0 | activation |
| 14.1687209663 | auc\_reaction\_6\_\_i | 1.0 | noncompetitive |
| 14.5760196595 | sigma\_reaction\_1\_\_i | 1.0 | noncompetitive |
| 15.5231714601 | auc\_reaction\_4\_\_i | 1.0 | noncompetitive |
| 19.9559223957 | tau\_reaction\_6\_\_i | 3.0 | noncompetitive |
| 21.843719459 | auc\_reaction\_7\_\_i | 10.0 | activation |
| 23.0486912381 | auc\_reaction\_5\_\_i | 10.0 | activation |
| 23.2304394063 | tau\_reaction\_1\_\_i | 1.0 | noncompetitive |
| 23.3382215442 | tau\_reaction\_4\_\_i | 3.0 | noncompetitive |
| 24.0765818916 | auc\_reaction\_3\_\_i | 10.0 | activation |
| 25.1474187565 | auc\_reaction\_8\_\_i | 3.0 | noncompetitive |
| 33.5811218567 | sigma\_reaction\_8\_\_i | 10.0 | noncompetitive |
| 38.1945189914 | auc\_reaction\_6\_\_i | 3.0 | noncompetitive |
| 40.8034244977 | sigma\_reaction\_6\_\_i | 10.0 | noncompetitive |
| 41.0020200607 | tau\_reaction\_8\_\_i | 10.0 | noncompetitive |
| 44.058963484 | auc\_reaction\_1\_\_i | 1.0 | noncompetitive |
| 44.823603692 | sigma\_reaction\_1\_\_i | 3.0 | noncompetitive |
| 45.7176764925 | auc\_reaction\_4\_\_i | 3.0 | noncompetitive |
| 48.7149941671 | sigma\_reaction\_4\_\_i | 10.0 | noncompetitive |
| 62.452267257 | tau\_reaction\_6\_\_i | 10.0 | noncompetitive |
| 66.3228479709 | auc\_reaction\_8\_\_i | 10.0 | noncompetitive |
| 71.2946530546 | tau\_reaction\_1\_\_i | 3.0 | noncompetitive |
| 79.3664324736 | tau\_reaction\_4\_\_i | 10.0 | noncompetitive |
| 115.148638071 | auc\_reaction\_6\_\_i | 10.0 | noncompetitive |
| 134.99849179 | auc\_reaction\_1\_\_i | 3.0 | noncompetitive |
| 151.506477329 | sigma\_reaction\_1\_\_i | 10.0 | noncompetitive |
| 151.783559058 | auc\_reaction\_4\_\_i | 10.0 | noncompetitive |
| 240.859381379 | tau\_reaction\_1\_\_i | 10.0 | noncompetitive |
| 455.780151147 | auc\_reaction\_1\_\_i | 10.0 | noncompetitive |
