# Supplementary figures and images for: TIde: a software for the systematic scanning of drug targets in kinetic network models
Source: BMC Bioinformatics. 2009 Oct 19;10:344. doi: 10.1186/1471-2105-10-344 (PMC2773792; doi:10.1186/1471-2105-10-344)

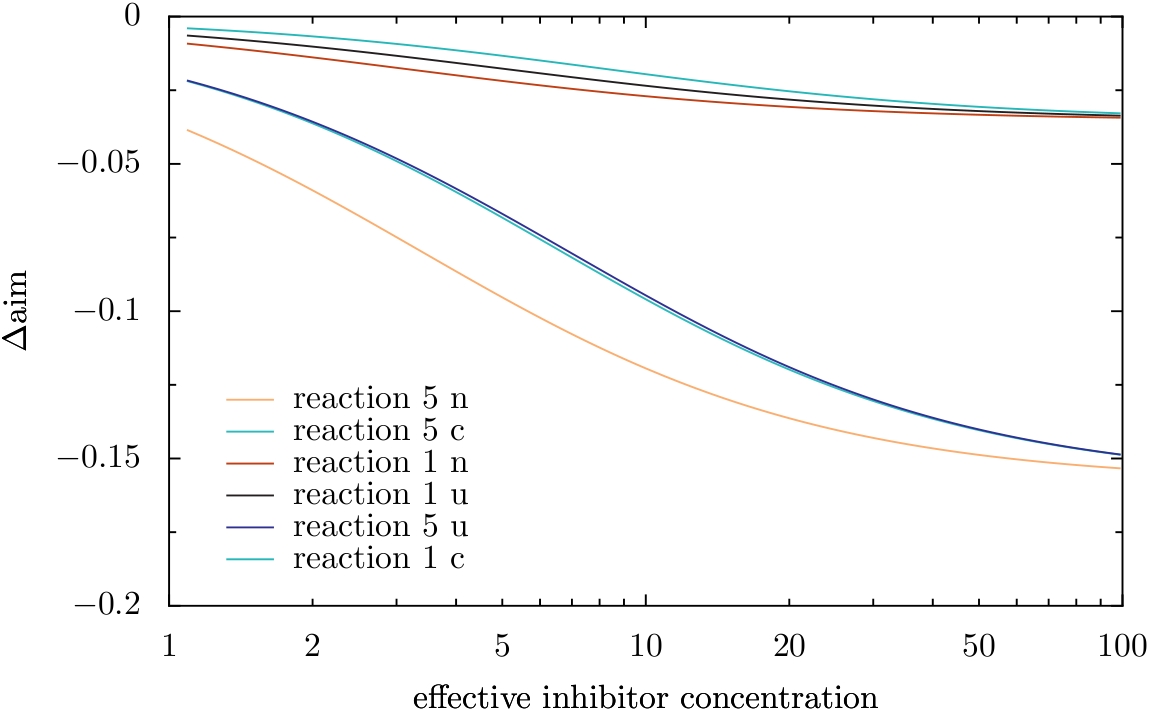

Supplement: Additional file 2 — TIde-1.2.1 source code. Contains the packed python source code of our tool. [file 1471-2105-10-344-S2.ZIP › TIde-1.2.1/documentation/pdfplot.jpeg]
